# Survival Time and Prognostic Factors of Mortality among Patients with Acquired Immunodeficiency Syndrome in North-East Peninsular Malaysia

**DOI:** 10.21315/mjms2019.26.4.8

**Published:** 2019-08-29

**Authors:** Hamiza Ngah, Suhaily Mohd Hairon, Najib Majdi Yaacob, Haniah Yusoff

**Affiliations:** 1 Department of Community Medicine, School of Medical Sciences, Universiti Sains Malaysia, Kubang Kerian, Kelantan, Malaysia; 2 Unit of Biostatistics & Research Methodology, School of Medical Sciences, Universiti Sains Malaysia, Kubang Kerian, Kelantan, Malaysia; 3 HIV/STI/Hep C Unit, Kelantan State Health Department, Ministry of Health Malaysia, Kelantan, Malaysia

**Keywords:** HIV/AIDS, median survival time, survival rate, prognostic factors

## Abstract

**Background:**

Death resulting from the acquired immunodeficiency syndrome (AIDS) is a worldwide concern. This study is aimed at determining the overall median survival time, and the prognostic factors of mortality among AIDS-infected patients in North-East Peninsular Malaysia.

**Methods:**

In 2018, a retrospective cohort study stretching from January to April was conducted. This study involved a review of data obtained from the National AIDS Registry. A total of 1,073 AIDS cases diagnosed from 1 January 2010 to 31 December 2014 were selected, and follow-up procedures were conducted until 31 March 2015 (a 3-month follow-up). The Kaplan-Meier plot and Cox’s proportional hazard regression were used for data analyses.

**Results:**

564 (52.5%) patients died due to AIDS, while the remaining 509 (47.4%) were censored. The overall median survival time was 11 months. The probability of survival in 1-year, 2-year, 3-year, 4-year and 5-year periods were 49.1%, 47.8%, 47.3%, 47.0% and 46.7%, respectively. Multiple Cox regression revealed that the significant prognostic factors were age 30–49 years [adjusted hazard ratio (Adj. HR) 1.57; 95% CI: 1.14, 2.16; *P* = 0.006], male (Adj. HR 1.39; 95% CI: 1.07, 1.79; *P* = 0.012), unemployed (Adj. HR 1.40; 95% CI: 1.12, 1.75; *P* = 0.003) and HIV-TB co-infection (Adj. HR 1.78; 95% CI: 1.37, 2.31; *P* < 0.001).

**Conclusion:**

The overall median survival time among AIDS patients in North-East Peninsular Malaysia was revealed to be short, in comparison to the other studies. The chances for survival can be improved with more emphasis on early detection (to ensure early treatment) and social support, particularly for HIV-TB co-infected patients, as well as for younger and unemployed patients.

## Introduction

The term acquired immunodeficiency syndrome (AIDS) refers to the most advanced stages of human immunodeficiency virus (HIV) infection. Globally, AIDS represents a major threat to public health (1). Since 2010, there has been a decline in the occurrence of AIDS-related deaths. This is mainly attributed to a substantial decrease in recorded cases in sub-Saharan Africa (53% reduction). In the Asia-Pacific region, AIDS-related deaths declined by 39% between 2010 and 2017. This is indicative of the rapid pace of treatment scale-up in the region (2).

In Malaysia, out of the 23,717 cases of AIDS reported between 1989 and 2016, 18,827 deaths were recorded. In 2016 alone, 7,000 Malaysian AIDS-related deaths were recorded (3). The survival rate of Malaysian AIDS patients improved markedly with the introduction of the anti-retroviral treatment (ART) in 1996 (4). Among Malaysian HIV-infected patients on the ART, it was reported that 72.7% survived with the viral load at ≤ 50 copies/mL. The median survival time was recorded as 130.9 months (5). In a separate study, a slightly more extended median survival time was reported (4).

According to literature relevant to this subject, the introduction of the ART during the late ‘90s led to an improvement in the survival rate of AIDS patients. Similar results were attained through recently conducted studies in the western world (6, 7). The survival odds of AIDS patients, however, depend on many other factors. These factors range from sociodemographic issues to clinical characteristics (6).

Identifying the prognostic factors of death among AIDS patients, will pave the way for improved HIV/AIDS prevention, care and treatment programmes in Malaysia. This, in turn, can guide clinicians towards achieving the goals of the National Strategic Plan of Ending AIDS 2016–2030 (NSPEA). The goals of the NSPEA are (i) to test 90% of the key affected population and record the results, (ii) to ensure that 90% of people living with HIV (PLHIV) receive the ART and (iii) to achieve viral suppression for 90% of people on ART. Due to differences in socio-demography and clinical characteristics, the heterogeneity of findings on prognostic factors of death from other studies, may not be adaptable to the situation in North-East Malaysia, and Malaysia as a whole. Therefore, it is critical that the AIDS patient survival time analysis be conducted in such a manner, that it meets local and region-wide information needs. The primary objective of this study is to determine the survival time, survival rate and prognostic factors of mortality among AIDS-infected patients in North-East Peninsular Malaysia.

## Methods

### Study Location

This study, which stretched from January to April, 2018, was conducted at the HIV unit of the Kelantan State Health Department, Malaysia. North-East Peninsular Malaysia consists of three states: Kelantan, Terengganu and Pahang. The state of Kelantan is bounded by Thailand in the north, the South China Sea in the northeast, Terengganu in the east, Pahang in the south and Perak in the west.

### Study Design

A retrospective cohort study was used to enrol AIDS patients with the period of diagnosis recorded as 1 January 2010 until 31 December 2014. The investigation for each AIDS case extended from the date of diagnosis, to the date of event, or whichever came first. The data on AIDS cases in Kelantan was sourced from the National AIDS Registry (NAR). Established in 2009, this surveillance system is managed by HIV units operating in all Malaysian states, and coordinated by the HIV section, the Disease Control Division, and the Malaysian Ministry of Health. This system, which is an internet-based registry, gathers data on confirmed HIV, AIDS and AIDS death cases from government and private Malaysian health facilities. This registry covers data related to the demographic background, the risk factor, the date of diagnosis, contact information, AIDS-related symptoms, as well as AIDS-related deaths. This data is accessible to authorised public health officers equipped with a registered username and password. This serves to ensure that confidentiality is preserved. A nationwide system, the NAR links the HIV units in each District Health Office, State Health Department, and Disease Control Division under the National Department of Public Health, through the network of the Ministry of Health (MOH) of Malaysia. All AIDS cases registered in NAR based on Case Definition for Infectious Diseases in Malaysia were selected, and follow-up operations were conducted until 31 of March, 2015. The samples for this study are (i) AIDS cases (8) diagnosed from 1 January 2010 until 31 December 2014, and (ii) Kelantan residents. Patients aged less than 12 years and patients with incomplete data were excluded.

### Sample Size

Two median survival time formulas, in the power and sample size software (9), were used to determine the sample size. The calculation for sample size was based on the variable age at the time of diagnosis (10). With type I error (alpha) of 0.05 (two tailed), type II error of 20% (power of study 80%), median survival time among the younger age group of 61 months, estimated median survival time among the older age group of 45 months, ratio between younger to older age groups of 2, accrual time of 60 months, additional follow-up time of 3 months and missing data of 20%, the required sample size calculated was 1,371. As the number of patients in the registry within the time period was smaller than the calculated sample size, no sampling method was applied. Therefore, all available cases that fulfilled the study criteria were included in the study.

### Data Collection

Data collection was performed with the use of the Proforma checklist as a guide for data extraction. The information required included age at diagnosis (in years), sex (male or female), ethnicity (Malay or non-Malay), marital status (single or married), occupation, HIV-TB co-infection (no or yes), date of diagnosis, date of death and cause of death. Only data relevant to this study were extracted. To ensure confidentiality, all data extraction processes were conducted at the HIV unit of the Kelantan State Health Department.

### Statistical Analysis

Data from the registry were extracted, conveyed to the Microsoft Excel file, and subsequently exported to the SPSS statistics version 24 for statistical analysing. Exploratory data analysis was performed to check for missing data, and normality distribution of numerical variables. Descriptive statistics were conducted to summarise the socio-demographic and clinical characteristics. Numerical variables were presented as mean (SD) or median (IQR), and categorical variables were presented as number (*n*) and percentage (%)

The event of interest for this study is death due to AIDS. Patients who did not suffer the event of interest by the 31 of March 2015, were considered censored. The Kaplan Meier survival analysis was used to estimate the overall median survival. Lifetable was applied to calculate the cumulative probability of survival rate with AIDS, and presented in percentage form. Univariable, followed by multivariable Cox regression analysis, was performed to identify the important prognostic factors of death. During univariable analysis, variables with *P* < 0.25 were selected to be included in the multivariable analysis. During multivariable analysis, the backward likelihood ratio (LR), forward LR and Stepwise methods were used to obtain the preliminary main effect model. The multiplicative method was used to check all possible two-way interactions. The hazard function plot and the log-minus-log plot were used to examine the assumption of proportional hazard. The final model was presented as crude and adjusted hazard ratio (HR), 95% confidence interval (CI), Wald statistics and *P*-value. The level of significance α was set at 0.05 (two tailed), with the predetermined power of study at 80%.

## Results

A total of 1,295 reported and registered cases in the National AIDS Registry were studied. However, due to incomplete data (data missing more than one variable) and duplication, 206 cases were excluded from this study. Subsequent to an eligibility examination on the remaining 1,089 cases, 11 patients were excluded as they were not Kelantan residents, while a further 5 were excluded as they were less than 12 years of age. Among the 1,073 patients, the proportion of AIDS-related deaths amounted to 52.6% (*n* = 564). The characteristics profile of the respondents can be observed in Table 1.

**Figure 1:**
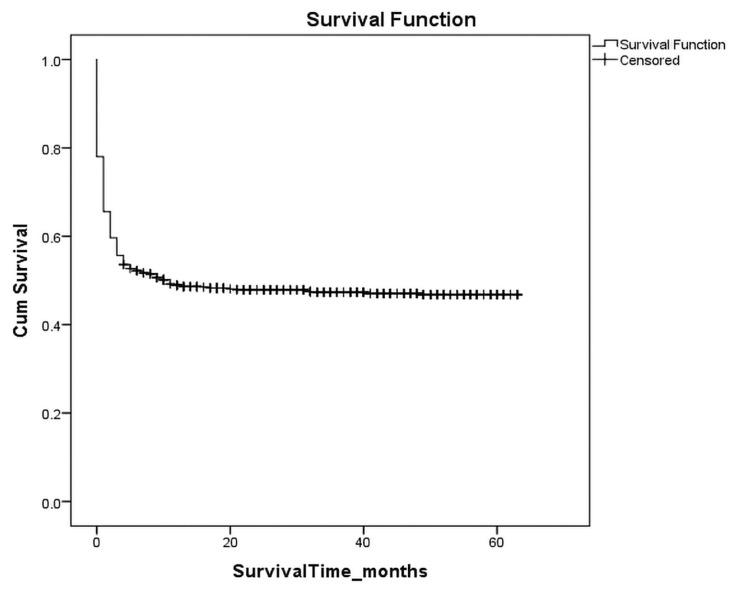
Kaplan-Meier estimate of overall median survival among AIDS patients in Kelantan

The Kaplan-Meier survival analysis revealed the overall median survival time among Kelantanese AIDS patients to be 11 months (Figure 1). The probability of survival in 1-year, 2-year, 3-year, 4-year and 5-year periods were 49.1%, 47.8%, 47.3%, 47.0% and 46.7%, respectively. The results attained through the Cox univariate analysis are presented in Table 2. According to the univariable analysis, patients who are within the age of 30 years to 49 years and ≥ 50 years, unemployed, HIV-TB co-infected and with an unknown status of HIV-TB co-infection, had a *P*-value of < 0.25. As such, these variables were included in the multivariate analysis. The results from the final multivariate model are displayed in Table 3. Four significant prognostic factors of death among Kelantanese AIDS patients were identified: 30 years to 49 years of age, males, unemployment and HIV-TB co-infection.

The hazard of death increased by 40% for unemployed patients in comparison to employed patients (Adj. HR 1.40; 95% CI: 1.12, 1.75; *P* = 0.003). For patients who were diagnosed with HIV co-infection with tuberculosis (HIV-TB), and patients with an unknown status of HIV-TB co-infection, the vulnerability to death is 78% higher than those without the HIV-TB co-infection (Adj. HR 1.78; 95% CI: 1.37, 2.31; *P* < 0.001; and Adj. HR 1.78; 95% CI: 1.49, 2.14; *P* < 0.001). Patients within the age of 30 years–49 years (Adj. HR 1.57; 95% CI: 1.14, 2.16; *P* = 0.006) and male patients (Adj. HR 1.39; 95% CI: 1.07, 1.79; *P* = 0.012), were also identified as significant prognostic factors of mortality, with an increased vulnerability to death of 57% and 39%, respectively.

## Discussion

The National AIDS Registry is an established public health surveillance system that records all HIV and AIDS cases, as well as AIDS-related deaths. Dedicated liaison officers monitor and update the status of all registered cases, at the state and national levels. Hence, the data on AIDS patients in Kelantan, used for this study, can be deemed reliable and applicable.

This study was focused on determining the overall median survival time, from the diagnosis of AIDS to death, regardless of any AIDS-defining disease. The overall median survival time ascertained was 11 months, with the probability of survival of 49.1% in year 1, 47.8% in year 2, 47.3% in year 3 and 46.0% in year 4. In comparison to data compiled through studies conducted in Western countries, the survival rate of patients in our population was observed to be lower (6, 7). It has been demonstrated, that the use of ART to curb progression from HIV to AIDS, enhances the potential for survival. The disparity in survival time, between AIDS patients in North-East Peninsular Malaysia, and patients in other countries, can partly be put down to differences in ART coverage.

In Malaysia, efforts to expand the availability and accessibility of ART began with the provision of free monotherapy in 2001. In 2002, Malaysia adopted a highly active ART policy involving four strategies: (i) the provision of free dynamic ART to all HIV patients with a CD4 count of below 400, (ii) the lowering of HIV drug prices through negotiations with highly active ART patent holders, (iii) the encouragement of local HIV drug production and (iv) due consideration, with regards to the application of ‘Right of Government’ under the Patent Act 1983, to ensure the sustainable supply of HIV drugs (3).

A study conducted in Guangxi, China, documented a strong association between the survival rate among AIDS patients, and the use of ART. This study highlighted the use of AZT monotherapy and combination therapy to reduce the risk of mortality (11). Similarly, Zhang et al. (12) reported a rise in median survival time among AIDS patients subjected to ART. Thus, it can be surmised, that ART has the capacity to extend the lifespan of AIDS patients.

The HIV/AIDS programme, which covers voluntary HIV screening and testing, facilitates the early detection and treatment of HIV/AIDS. This serves to improve the survival rate among AIDS patients (13). With this programme, we can be considered on the right track towards realising the target (zero AIDS-related death count) of our National Strategic Plan of Ending AIDS (14).

This study uncovered that the age of diagnosis between 30 years to 49 years, the male gender, unemployment, HIV-TB co-infection and the unknown status of HIV-TB co-infection, are significantly associated to the vulnerability to death among AIDS patients in Kelantan. On the other hand, ethnicity, educational level and marital status were not observed to be significant prognostic factors of death.

This study revealed that the threat of death among middle aged patients (30 years old–49 years old), is more pronounced than that for those in the older age. This finding is in agreement to those reported in several other studies (5, 10). Many studies also identified age at diagnosis an important prognostic factor of death among HIV/AIDS patients. These studies linked the late diagnosis of AIDS to a shorter survival time (5, 15). The late diagnosis of HIV (and the consequently delayed treatment), is a major contributor towards morbidity and mortality (16). May et al. (17) concluded that the increase in mortality with the increase in the age of diagnosis, is related to the immune response to the CD4 cell count level, and the viral load level of the individual.

For the most part, males are deemed the predominant group of HIV-infected individuals. This situation has been linked to the needle and syringe sharing behaviour among drug users, who are mostly male (18). According to the results from our study, the mortality rate is significantly higher for male patients than for female patients.

Our investigation revealed that the hazard of death is 1.4 times higher for unemployed patients, in comparison to employed patients. Among the determinants of progression to AIDS is occupational status (15, 19). Several studies have revealed unemployment to be a predictor for risk of mortality among AIDS patients (20, 21). Accessibility and affordability of treatment are closely related to an individual’s employment status. Thus, it is to be expected that the survival period among employed AIDS patients, is longer than that among unemployed AIDS patients (22). Additionally, employed individuals will have the capacity to select and purchase nutritious food and/or supplements. This can help to boost their immunity, and consequently extend their survival period (21).

Tuberculosis is the most common life-threatening opportunistic infection in HIV patients (23). Twenty-six percent of AIDS-related deaths are attributed to tuberculosis, with 99% of such deaths occurring in developing countries (24). According to the results attained through our study, AIDS patients in Kelantan with HIV-TB co-infection are 1.78 times more likely to succumb to mortality, than patients who are without tuberculosis co-infection. Other studies revealed that the use of co-trimoxazole prophylaxis and access to rifampicin, can extend the survival period of patients with HIV-TB co-infection.

On the other hand, a rapid depletion of the CD4 cell count can shorten the survival period of such patients (24, 25).

### Strengths and Limitations

To the best of our knowledge, this is the first study conducted on the survival rate of AIDS patients in Kelantan. The external validity of this study is reinforced by the fact that the data used was sourced from a high quality and reliable official database (the Malaysian National AIDS registry). The limitations encountered during this undertaking include the retrospective nature of the patient’s follow-up process, missing or incomplete information in the secondary data, under-reporting or reporting delays in the surveillance system that resulted in an exceedingly late AIDS diagnosis.

## Conclusion

Identification of the significant prognostic factors, can present the health care provider with the required input, for re-designing the clinical management of high-risk patients. Additionally, knowledge of these prognostic factors can assist clinicians during efforts to convince patients with regards to the prognosis. This will go a long way towards improving the patients’ chances for survival, as well as their quality of life.

## Figures and Tables

**Table 1:** Profile of AIDS patients identified through the National AIDS Registry, 2010–2014 (*n* = 1,073)

Characteristics profile	*n* (%)	Censored	Death

		*n* (%)	*n* (%)
Age at diagnosis (years)	37.08 (7.37)*		
Age at diagnosis			
13–29	114 (10.6)	72 (63.2)	42 (36.8)
30–49	901 (84.0)	410 (45.5)	491 (54.5)
≥50	58 (5.4)	27 (46.6)	31 (53.4)
Sex			
Female	140 (13)	55 (39.3)	85 (60.7)
Male	933 (87.0)	454 (48.7)	479 (51.3)
Ethnicity			
Malay	939 (87.5)	439 (46.8)	500 (53.2)
Non-Malay	134 (12.5)	70 (52.2)	64 (47.8)
Marital status			
Single	668 (62.3)	323 (48.4)	345 (51.6)
Married	282 (26.3)	131 (46.5)	151 (53.5)
Divorced/widower	123 (11.5)	55 (44.7)	68 (55.3)
Occupation			
Unemployed	408 (38.0)	169 (41.4)	239 (58.6)
Employed	238 (22.2)	124 (52.1)	114 (47.9)
Others	427 (39.8)	216 (50.6)	211 (49.4)
Education level			
No formal education	100 (9.3)	50 (50.0)	50 (50.0)
Primary school	119 (11.1)	55 (46.2)	64 (53.8)
Secondary school	838 (78.1)	396 (47.3)	442 (52.7)
Higher education	16 (1.5)	8 (50.0)	8 (50.0)
HIV-TB			
Yes	99 (9.2)	29 (29.3)	70 (70.7)
No	674 (62.8)	381 (56.5)	293 (43.5)
Unknown	300 (28.0)	99 (33.0)	201 (67.0)

* Mean (SD)

**Table 2:** Prognostic factors of mortality by Simple Cox proportional hazards regression (*n* = 1,073)

Variables	Crude *b*	Crude HR (95% CI)	Wald statistic	*P*-value
Age at diagnosis				
13–29		1		
30–49	0.49	1.63 (1.19, 2.23)	9.10	0.003
≥50	0.47	1.59 (1.00, 2.54)	3.88	0.049
Sex				
Female		1		
Male	0.11	1.22 (0.97, 1.54)	2.87	0.090
Ethnicity				
Non-Malay		1		
Malay	0.10	1.11 (0.85, 1.44)	0.60	0.440
Marital status				
Married		1		
Single	−0.02	0.98 (0.81, 1.19)	0.03	0.861
Divorced/ widower	−0.03	1.07 (0.80, 1.42)	0.20	0.654
Occupation				
Employed		1		
Unemployed	0.30	1.35 (1.08, 1.69)	6.88	0.009
Others	0.07	1.07 (0.85, 1.34)	0.33	0.568
Education level				
No formal education		1		
Primary school	0.08	1.09 (0.75, 1.57)	0.19	0.664
Secondary school	0.02	1.03 (0.77, 1.37)	0.03	0.871
Higher education	0.03	1.03 (0.49, 2.17)	0.01	0.941
HIV-TB				
No		1		
Yes	0.60	1.83 (1.41, 2.38)	20.39	< 0.001
Unknown	0.60	1.83 (1.52, 2.19)	42.39	< 0.001

*b* = regression coefficient

**Table 3:** Prognostic factors of mortality by Multiple Cox proportional hazards regression (*n* = 1,073)

Variables	Adj. *b*	Adj. HR (95% CI)	Wald statistic	*P*-value
Age (year)				
13–29		1		
30–49	0.45	1.57 (1.14, 2.16)	7.58	0.006
≥50	0.44	1.55 (0.97, 2.47)	3.31	0.069
Sex				
Female		1		
Male	0.33	1.39 (1.07, 1.79)	6.30	0.012
Occupation				
Employed		1		
Unemployed	0.34	1.40 (1.12, 1.75)	8.74	0.003
Others	0.06	1.06 (0.83, 1.35)	0.21	0.648
HIV-TB				
No		1		
Yes	0.58	1.78 (1.37, 2.31)	18.22	< 0.001
Unknown	0.58	1.78 (1.49, 2.14)	38.60	< 0.001

Adj. *b* = Adjusted regression coefficient; backward LR stepwise Cox proportional hazard regression model was applied; Interaction term were not significant in all factors; Proportional hazard assumption by hazard function plot and log-minus-log plot were checked and assumptions were met
